# Long-term clinical outcomes in type 1 Gaucher disease following 10 years of imiglucerase treatment

**DOI:** 10.1007/s10545-012-9528-4

**Published:** 2012-09-14

**Authors:** Neal J. Weinreb, Jack Goldblatt, Jacobo Villalobos, Joel Charrow, J. Alexander Cole, Marcelo Kerstenetzky, Stephan vom Dahl, Carla Hollak

**Affiliations:** 1 University Research Foundation for Lysosomal Storage Diseases, Inc, Northwest Oncology Hematology Associates PA, 8170 Royal Palm Boulevard, Coral Springs, FL 33065 USA; 2 Genetic Services & Familial Cancer Program of Western Australia, School of Pediatrics and Child Health, University of Western Australia, Perth, WA Australia; 3 Universidad Central de Venezuela, Caracas, Venezuela; 4 Division of Genetics, Birth Defects and Metabolism, Ann and Robert H. Lurie Children’s Hospital of Chicago, Chicago, IL USA; 5 Genzyme, a Sanofi company, Cambridge, MA USA; 6 Instituto de Medicina Integral Professor Fernando Figueira, Recife, Pernambuco Brazil; 7 Klinik für Innere Medizin, St. Franziskus-Hospital, Cologne, Germany; 8 Department of Internal Medicine, Division of Endocrinology and Metabolism, Academic Medical Center, University of Amsterdam, Amsterdam, The Netherlands

## Abstract

**Objective:**

We studied the effect of long-term alglucerase/imiglucerase (Ceredase®/Cerezyme®, Genzyme, a Sanofi company, Cambridge, MA, USA) treatment on hematological, visceral, and bone manifestations of Gaucher disease type 1 (GD1).

**Methods:**

The International Collaborative Gaucher Group (ICGG) Gaucher Registry identified GD1 patients treated with alglucerase/imiglucerase who had dose and clinical data at first infusion and after 10 years of follow-up. Data for hemoglobin, platelet count, organ volumes, bone pain, and bone crisis were analyzed. Tests of the null hypothesis (no change from first infusion to 10 years) were performed using* t* tests for within-patient absolute change in continuous measurements and McNemar/chi-square tests for change in distributions using categorical values. An alpha level of 0.05 designated statistical significance.

**Results:**

As of October 2011, 557 nonsplenectomized and 200 splenectomized patients met the inclusion criteria. The majority of GD1 patients had at least one N370S allele. Compared with nonsplenectomized patients at first infusion, splenectomized patients had lower percentages of anemia (26.0 % vs. 42.8 %) and thrombocytopenia (14.2 % vs. 76.3 %), similar percentages of moderate or severe hepatomegaly (81.2 % vs. 80.0 %), and higher percentages of bone pain (88.9 % vs. 52.4 %) and bone crises (38.3 % vs. 16.0 %). After 10 years, both groups showed significant (*p* < 0.05) improvements in mean hemoglobin levels, platelet count, liver, and spleen (nonsplenectomized) volumes, and bone crises. Initial dosing in both groups ranged from <15 U/kg to ≤90 U/kg every 2 weeks. After 10 years, the majority was receiving 15 to ≤45 U/kg every 2 weeks.

**Conclusion:**

Ten years of imiglucerase treatment results in sustainable improvements in all GD1 parameters.

**Electronic supplementary material:**

The online version of this article (doi:10.1007/s10545-012-9528-4) contains supplementary material, which is available to authorized users.

## Introduction

Gaucher disease (GD) is an autosomal recessive lysosomal storage disorder that results from loss of function of acid β-glucosidase (EC 3.2.1.45; lysosomal glucocerebrosidase) due to mutations in the glucocerebrosidase gene, *GBA1* (Grabowski et al. 2010). Deficient glucocerebrosidase activity leads to accumulation of the enzyme’s substrate, glucocerebroside, in tissue macrophages and to displacement of normal cells in tissues of multiple organs, such as liver, spleen, and bone marrow (Charrow et al. 2000). Clinical manifestations of GD include hematological abnormalities (thrombocytopenia, anemia, leukopenia), splenic and hepatic enlargement (with associated dysfunction), skeletal disease (chronic bone pain, acute bone crises, defective bone mineralization, infarction, osteonecrosis, osteolysis, and pathological fractures) (Charrow et al. 2000; Weinreb et al. 2008), growth retardation (Kaplan et al. 2006), and decreased health-related quality of life (HR-QoL) (Weinreb et al. 2007). The traditional classification of GD recognizes three clinically distinct forms: type 1 (nonneuronopathic, OMIM #230800), type 2 (acute neuronopathic, OMIM #230900), and type 3 (chronic neuronopathic, OMIM #231000). GD type 1 (GD1) is differentiated from GD2 and GD3 by the absence of overt, early-onset neurological signs and symptoms (Grabowski et al. 2010). However, distinct neurological symptoms such as peripheral neuropathy and Parkinson’s disease may occur in GD1 (Biegstraaten et al. 2008).

GD1 was the first lysosomal storage disease to be successfully treated with enzyme therapy, initially in 1991 with placental-derived glucocerebrosidase, (alglucerase, Ceredase®, Genzyme Corporation,^1^ Cambridge, MA, USA), and beginning in 1994, with recombinant glucocerebrosidase, (imiglucerase, Cerezyme®, Genzyme). Imiglucerase is highly effective in reversing visceral and hematologic manifestations of GD as well as some aspects of skeletal disease (Andersson et al. 2005; Barton et al. 1991; Charrow et al. 2007; Grabowski et al. 1998; Sims et al. 2008; Weinreb et al. 2008; Weinreb et al. 2002; Wenstrup et al. 2007) over a wide range of doses (Grabowski et al. 2009).

Evaluation of hematological and organ responses and skeletal manifestations is the standard minimum criterion for evaluating efficacy of treatment for GD1 in both clinical trials and in everyday practice. This report used observational data from the International Collaborative Gaucher Group (ICGG) Gaucher Registry to analyze responses of GD1 patients at first infusion and after 10 years of imiglucerase treatment. We hypothesized that imiglucerase therapy would demonstrate a sustained benefit, even after 10 years of treatment.

## Materials and methods

### International Collaborative Gaucher Group (ICGG) Gaucher Registry

The ICGG Gaucher Registry was launched in 1991 to track clinical, demographic, genetic, biochemical, and therapeutic characteristics of patients with GD throughout the world, irrespective of disease severity, treatment status, or treatment choice (Charrow et al. 2000). Governance and scientific direction is provided by an international group of physician experts in GD, with operational support from Genzyme, a Sanofi company (Cambridge, MA, USA). With Institutional Review Board (IRB)/Ethics Committee approvals and after obtaining informed patient consent, physicians from 62 countries voluntarily submitted deidentified data on more than 6,000 patients, capturing 54,000 patient years of follow-up.

### Study population

We identified all GD1 patients in the ICGG Gaucher Registry as of October 2011 who received imiglucerase (Cerezyme®, Genzyme) or alglucerase (Ceredase®, Genzyme), which are therapeutically equivalent treatments (Grabowski et al. 1995). In the report presented here, the term imiglucerase is used to denote patients who received either alglucerase or imiglucerase. Inclusion criteria for the study data set were patients with GD1 who had dose and clinical data at first imiglucerase infusion (baseline) and after 10 years of therapy. Within the study cohort, subsets of patients were identified with clinical data for hemoglobin, platelet count, liver volume, spleen volume, bone pain, and bone crisis at treatment initiation (baseline) and after 10 years of follow-up. Patients without clinical data at baseline and after 10 years were excluded.

Because splenectomy is known to have an immediate and persistent influence on GD pathophysiology and patient phenotype, osteonecrosis (Deegan et al. 2011; Mistry et al. 2009), and hematological response (Cox et al. 2008; Hughes et al. 2007), data were further analyzed by splenectomy status. Patients were stratified into nonsplenectomized and splenectomized groups. Because the postprocedure phenotype after partial splenectomy is generally closer to that of patients with fully intact spleens than to those with total splenectomy, in this treatment response analysis, the small number of eligible partial splenectomy patients (*N* = 27) were included among the much larger number of patients with fully intact spleens (*N* = 557).

For this analysis, baseline was defined as a window of time ranging from 12 months prior to 1 month following the date of treatment initiation. The 10-year interval was defined as the time from 9.5 years to 10.5 years. Within each time window, whenever a patient had more than one clinical assessment date, the value associated with the date closest to the date of treatment initiation or the date closest to the 10-year interval was incorporated into the analysis. No data were included after the Cerezyme manufacturing interruption (25 June 2009). If patients switched to an alternative form of enzyme replacement therapy, their data were excluded from the analysis.

### Statistical analysis

Demographics and clinical parameters at treatment initiation and after 10 years (hemoglobin, platelet count, liver volume, spleen volume, bone pain, and bone crisis), and frequency of genotypes were characterized using descriptive statistics. Organ volumes, measured by magnetic resonance imaging (MRI), computed tomography (CT), or ultrasound (US), are reported in multiples of normal (MN) size predicted for body weight: approximately 2.5 % of body weight for liver, and 0.2 % for spleen (Elstein et al. 1997; Ludwig 1979). Clinical parameters were analyzed both as continuous measurements, and as categorical measurements using the following cut points:

- Anemia is defined according to age and gender norms for hemoglobin concentrations, as follows: <12 g/dl for males >12 years; <11 g/dl for females >12 years; <10.5 g/dl for children ages >2–12 years; <9.5 g/dl for children ages 6 months to 2 years; <10.1 g/dl for children younger than 6 months of age.
- Thrombocytopenia is categorized as mild or none (≥120 × 10^3^/mm^3^), moderate (60 to <120 × 10^3^/mm^3^), or severe (<60 × 10^3^/mm^3^).
- Splenomegaly (spleen volume in multiples of normal) is scored as mild or none (≤5), moderate (>5 to ≤15), or severe (>15).
- Hepatomegaly (liver volume in multiples of normal) is scored as mild or none (≤1.25), moderate (>1.25 to ≤2.5), or severe (>2.5).
- Bone pain is defined as being present if the patient reported this event as occurring in the 30-day interval before the medical visit.
- Bone crisis was defined as present if the patient reported this event at the time of the medical visit. The ICGG Gaucher Registry defines bone crisis as: “Pain with acute onset that requires immobilization of the affected area, narcotics for the relief of pain, and may be accompanied by 1 or more of the following: periosteal elevation: elevated white blood cell count, fever, or debilitation > 3 days.”
- Doses used were those reported at first infusion and at 10 years.

For analysis of continuous values, we calculated both the within-patient absolute change and percentage change from treatment initiation to 10 years. For categorical analysis, we produced shift tables representing change in the distribution of values from first infusion to 10 years. The normality of the distributions was tested, and paired * t* tests were performed. Tests of the null hypotheses (i.e. no change from first infusion to 10 years) were calculated using the paired * t* test for the within-patient absolute change in continuous measurements, the McNemar test for binary variables, and the chi-square test when a variable had more than two categories. An alpha level of 0.05 denotes statistical significance.

## Results

As of October 2011, 757 GD1 patients enrolled in the ICGG Gaucher Registry met the study inclusion criteria of receiving imiglucerase and having both clinical data and dose data at first infusion (baseline) and after 10 years of follow-up. Of these, 226 patients received imiglucerase only, and 531 started treatment with alglucerase and transitioned to imiglucerase. Of the overall patient cohort, 200 were splenectomized and 557 were not.

### Nonsplenectomized patients

The demographic characteristics of nonsplenectomized GD1 patients are shown in Table 1. Most patients were diagnosed early in life (median age at diagnosis: 11 years). The median age for first imiglucerase infusion was not until 24 years. Genotypes were reported for approximately 89 % of nonsplenectomized patients (Table 2). Nearly all (92 %) had at least one N370S allele (27 % homozygous, 65 % heteroallelic). Among patients with available baseline data, prior to starting imiglucerase treatment, 42.8 % of nonsplenectomized patients were anemic, 76.3 % had moderate or severe thrombocytopenia, 86.9 % had moderate or severe splenomegaly, 80.0 % had moderate or severe hepatomegaly, 52.4 % reported bone pain, and 16.0 % reported bone crises (Table 4; Supplementary Figs. 1–6).

**Table 1:** Demographic characteristics of nonsplenectomized and splenectomized patients with type 1 Gaucher disease

	Nonsplenectomized^a^	Splenectomized
Patients Enrolled	*N* = 557	*N* = 200
Sex,* N* (%)	*N* = 557	*N* = 200
Males	263 (47)	80 (40)
Females	294 (53)	120 (60)
Age at Diagnosis^**b**^ (years)	*N* = 554	*N* = 196
Median (25th, 75th)	11 (5, 30)	10 (4, 23)
Mean (SD)	18 (16)	15 (13)
Min, Max	<0^c^, 75	0, 53
Age at Diagnosis^b^,* N* (%)	*N* = 554	*N* = 196
Prenatal^c^ to <10 Years	261 (47)	95 (48)
10 to <20 years	76 (14)	39 (20)
20 to <30 years	81 (15)	33 (17)
30 to <40 years	60 (11)	18 (9)
40 to <50 years	50 (9)	9 (5)
50 to <60 years	20 (4)	2 (1)
60 to <70 years	5 (1)	0
≥70 years	1 (<1)	0
Age at First infusion (years)	*N* = 557	*N* = 200
Median (25th, 75th)	24 (9, 42)	38 (26, 49)
Mean (SD)	26 (19)	37 (16)
Min, Max	0, 75	3, 74
Age at Last Follow-up (years)	***N*** = 557	*N* = 200
Median (25th, 75th)	37 (23, 56)	54 (40, 63)
Mean (SD)	40 (19)	52 (16)
Min, Max	13, 87	18, 87

*SD* standard deviation

^a^Includes 27 patients with partial splenectomy

^b^Patients with no diagnosis date or with diagnosis date earlier than 1 year prior to birth were excluded from the analysis

^c^Diagnosed prenatally

**Table 2:** Frequency of genotype categories of nonsplenectomized and splenectomized patients with type 1 Gaucher disease

Genotype, *N* (%)	Nonsplenectomized^c^ (*N* = 493)	Splenectomized (*N* = 189)
N370S/N370S	132 (27)	25 (13)
N370S/L444P	106 (22)	37 (20)
N370S/rare allele^a^	93 (19)	26 (14)
N370S/?^b^	52 (11)	27 (14)
N370S/84GG	51 (10)	30 (16)
N370S/IVS2 + 1	17 (3)	10 (5)
L444P/rare allele^a^	11 (2)	8 (4)
Rare allele^a^/rare allele^a^	11 (2)	10 (5)
L444P/L444P	8 (2)	4 (2)
L444P/?^b^	3 (1)	7 (4)
D409H/rare allele^a^	2 (<1)	–
? ^b^/? ^b^	2 (<1)	1 (1)
L444P/D409H	1 (<1)	–
D409H/D409H	1 (<1)	–
84GG/rare allele^a^	1 (<1)	2 (1)
IVS2 + 1/rare allele^a^	1 (<1)	1 (1)
Rare allele^a^/?^b^	1 (<1)	–
84GG/?^b^	–	1 (1)

^a^Rare allele is defined as known allele that is not N370S, L444P, IVS2 + 1, D409H, or 84GG

^b^Results of genotype test did not match any tested mutations

^c^Includes 27 patients with partial splenectomy

Changes in hematological, visceral organ, and skeletal characteristics reported at first infusion and after 10 years of treatment are shown in Table 3. For mean hemoglobin concentration (g/dl), there was a significant improvement observed at 10 years (11.2 increased to 13.6; *P* < 0.0001) compared with the start of treatment. Approximately 88 % of nonsplenectomized patients who were anemic at first infusion were no longer so after 10 years of treatment (Table 4, Supplementary Fig. 1a).

**Table 3:** Change in clinical parameters from initiation of imiglucerase to 10 years following first infusion in nonsplenectomized and splenectomized patients with type 1 Gaucher disease

Nonsplenectomized^a^				Splenectomized		
Parameter	First Infusion	10 Years Following First Infusion	Calculated Change	First Infusion	10 Years Following First Infusion	Calculated Change
Hemoglobin (g/dl)						
No.	376	376	376	131	131	131
Mean (SD)	11.2 (1.7)	13.6 (1.6)	2.4 (1.8)	11.9 (1.7)	13.4 (1.6)	1.5 (1.8)
Median	11.2	13.6	2.4	12.0	13.5	1.4
Min, Max	6.7, 16.2	8.8, 17.9	−3.0, 7.9	6.8, 16.9	6.9, 17.6	−5.0, 7.4
* P* value*			<0.0001			<0.0001
Platelet Count (x 10^3^/mm^**3**^ **)**						
No.	397	397	397	141	141	141
Mean (SD)	95.3 (52.8)	166.6 (59.7)	71.3 (51.9)	237.8 (125.5)	311.2 (98.3)	73.4 (126.0)
Median	87.0	169.0	71.0	218.0	307.0	68.0
Min, Max	16.0, 448.0	25.0, 433.0	−170.0, 219.0	6.0, 726.0	98.0, 644.0	−329.0, 464.0
* P* value*			<0.0001			<0.0001
Spleen Volume (Multiples of Normal)						
No.	107	107	107	–	–	–
Mean (SD)	19.4 (16.2)	5.2 (3.6)	−14.3 (15.0)	–	–	–
Median	15.9	3.7	−10.7	–	–	–
Min, Max	1.4, 91.8	1.2,15.7	−88.8,1.6	–	–	–
* P* value*			<0.0001	–	–	–
Liver Volume (Multiples of Normal)						
No.	105	105	105	32	32	32
Mean (SD)	1.8 (0.8)	1.0 (0.2)	−0.8 (0.7)	2.2 (1.0)	1.0 (0.24)	1.2 (0.91)
Median	1.7	1.0	−0.7	2.1	0.93	−0.97
Min, Max	0.6, 6.0	0.6, 1.9	−4.5, 0.2	0.7, 4.4	0.7, 1.7	−3.4, 0.1
* P* value*			<0.0001			<0.0001

*SD* standard deviation

^a^Includes 27 patients with partial splenectomy

*Tests of the null hypotheses (i.e. no change from first infusion to 10 years) were calculated using the paired t-test for the within-patient absolute change

Nonsplenectomized patients had a mean platelet count of 95.3 × 10^3^/mm^3^ at first infusion, which improved to a mean of 166.6 × 10^3^/mm^3^ after 10 years (*P* < 0.0001) (Table 3). At 10 years, the number of patients with severe thrombocytopenia decreased significantly (*P* < 0.001) (Supplementary Fig. 2a). The 397 patients eligible for platelet response analysis included 20 with partial splenectomies (5.0 %). Mean platelet count in these patients improved from 148 × 10^3^/mm^3^ at first infusion to 190 × 10^3^/mm^3^ after 10 years (data not shown). Nonsplenectomized GD1 patients (*N* = 107) demonstrated significant (*P* < 0.0001) improvement in spleen size over the 10 years of treatment. Mean spleen volume at first infusion was 19.4 MN and decreased to a mean of 5.2 MN after 10 years (Table 3). The number of patients with moderate and severe splenomegaly was significantly lower (*P* < 0.0001) after 10 years of imiglucerase therapy (Table 4, Supplementary Fig. 3). Only three partial splenectomy patients had sufficient data for inclusion in this analysis (mean spleen volume at first infusion: 9.0 MN; after 10 years: 3.5 MN; data not shown). There were also significant (*P* < 0.0001) reductions in liver size over 10 years of treatment (Table 3). At first infusion, the mean liver volume for nonsplenectomized patients was 1.8 MN and decreased to 1.0 MN after 10 years. The numbers of patients with moderate and severe hepatomegaly decreased significantly (*P* < 0.0001) after 10 years; no patients had severe hepatomegaly at this time point (Table 4, Supplementary Fig. 4a).

Imiglucerase treatment positively affected skeletal symptoms, as noted by decreased bone pain (Table 4, Supplementary Fig. 5a) and bone crises (Table 4, Supplementary Fig. 6a). For nonsplenectomized GD1 patients with bone pain data, 98/187 (52.4 %) reported bone pain prior to initiation of imiglucerase (Table 4, Supplementary Fig. 5a). Of these, 57.1 % (*n* = 56) no longer reported bone pain after 10 years of treatment. Of the 89 patients with no reported bone pain prior to initiation of imiglucerase, 75 (84.3 %) continued to be pain free when evaluated after 10 years of therapy. For nonsplenectomized patients with bone crisis data, 27/169 (16.0 %) had a report of bone crisis prior to initiation of imiglucerase. Of these, 92.6 % (*n* = 25) did not report a bone crisis after 10 years of treatment (Table 4, Supplementary Fig. 6a). None of the 142 patients who reported no bone crises prior to initiation of imiglucerase reported bone crises after 10 years of therapy. At initiation of treatment (Table 6), most patients were dosed in the middle range at either >15 to ≤45 U/kg every 2 weeks (*n* = 244 patients, 43.8 %) or >45 to ≤90 U/kg every 2 weeks (*n* = 198, 35.5 %). A smaller percentage of patients received low doses (≤15 U/kg every 2 weeks, *n* = 112, 20.1 %) and hardly any received high doses (>90 to ≤150 U/kg every 2 weeks, *n* = 3, <1 %). After 10 years of imiglucerase, most (82.2 %) were receiving imiglucerase doses in the middle range (>15 to ≤45 or >45 to ≤90 U/kg every 2 weeks), but now the majority (57.6 %) were receiving >15 to ≤45 U/kg every 2 weeks, reflecting shifts away from lower and higher dose groups.

**Table 4:** Change in clinical parameters from initiation of imiglucerase to 10 years following first infusion in non-splenectomized^a^ patients with type 1 Gaucher disease

	10 years following first infusion, *n* (%)				
First infusion	No	Yes	Total	*P* value*	
Anemia^b^					
No	210 (97.7)	5 (2.3)	215 (100)	<0.0001	
Yes	141 (87.6)	20 (12.4)	161 (100)		
Total	351 (93.4)	25 (6.6)	376 (100)		
Bone pain during past month					
Yes	56 (57.1)	42 (42.9)	98 (100)	<0.0001	
No	75 (84.3)	14 (15.7)	89 (100)		
Total	131 (70.1)	56 (29.9)	187 (100)		
Bone crises since last submission					
Yes	25 (92.6)	2 (7.4)	27 (100)	<0.0001	
No	142 (100.0)	0	142 (100)		
Total	167 (98.8)	2 (1.2)	169 (100)		
	10 years following first infusion, *n* (%)				
First infusion	Mild	Moderate	Severe	Total	*P* value**
Thrombocytopenia categories					
Mild (≥120)	88 (93.6)	6 (6.4)	0	94 (100)	<0.0001
Moderate (60 to <120)	175 (81.0)	40 (18.5)	1 (0.5)	216 (100)	
Severe (<60)	44 (50.6)	37 (42.5)	6 (6.9)	87 (100)	
Total	307 (77.3)	83 (20.9)	7 (1.8)	397 (100)	
Splenomegaly					
Mild (≤5)	14 (100.0)	0	0	14 (100)	<0.0001
Moderate (>5 to ≤15)	31 (83.8)	6 (16.2)	0	37 (100)	
Severe (>15)	21 (37.5)	34 (60.7)	1 (1.8)	56 (100)	
Total	66 (61.7)	40 (37.4)	1 (0.9)	107 (100)	
Hepatomegaly					
Mild (≤1.25)	21 (100.0)	0	0	21 (100)	<0.0001
Moderate (>1.25 to ≤2.5)	61 (92.4)	5 (7.6)	0	66 (100)	
Severe (>2.5)	8 (44.4)	10 (55.6)	0	18 (100)	
Total	90 (85.7)	15 (14.3)	0	105 (100)	

^a^Includes patients with partial splenectomy

^b^Anemia is defined according to age and gender norms for hemoglobin concentrations, as follows: <12 g/dl for males older than 12 years; < 11 g/dl for females older than 12 years; < 10.5 g/dl for children ages > 2–12 years; < 9.5 g/dl for children ages 6 months to 2 years; < 10.1 g/dl for children younger than 6 months

*Tests of the null hypotheses (i.e., no change from first infusion to 10 years) were calculated using the McNemar test

**Tests of the null hypotheses (i.e., no change from first infusion to 10 years) were calculated using the chi- square test

### Splenectomized patients

Demographic characteristics of splenectomized patients are shown in Table 1. As with patients with intact spleens, most splenectomized patients were diagnosed early in life (median age at diagnosis 10 years). The splenectomized group differed from the nonsplenectomized group in having proportionally more females (60 % vs. 53 %, respectively), a higher proportion of patients diagnosed from 10 to <30 years (37 % vs. 29 %), and older median ages at first imiglucerase infusion (38 years vs. 24 years) and at last follow-up (54 years vs. 37 years). Genotypes were reported for approximately 95 % of splenectomized patients (Table 2). The majority (82 %) had at least one N370S allele (13 % homozygous, 69 % heteroallelic) and were less likely to have N370S homozygosity and more likely to lack an N370S allele than patients with intact spleens. Prior to starting imiglucerase treatment, splenectomized patients had the following clinical status at baseline: 26.0 % were anemic, 14.2 % had moderate or severe thrombocytopenia, 81.2 % had moderate or severe hepatomegaly, 88.9 % reported bone pain, and 38.3 % reported bone crises (Table 5, Supplementary Figs. 1b, 2b, 4b, 5b, 6b). Compared with the nonsplenectomized group, there was a lower percentage of patients with anemia (26.0 % vs. 42.8 %) and thrombocytopenia (14.2 % vs. 76.3 %), a similar percentage with moderate or severe hepatomegaly (81.2 % vs. 80.0 %), and a higher percentage with bone pain (88.9 % vs. 52.4 %) and bone crises (38.3 % vs. 16.0 %).

**Table 5:** Change in clinical parameters from initiation of imiglucerase to 10 years following first infusion in splenectomized patients with type 1 Gaucher disease

Splenectomized patients					
	10 years following first infusion,* N* (%)				
First infusion	No	Yes	Total	*P *value*	*P* value**
Anemia^a^					
No	94 (96.9)	3 (3.1)	97 (100)	<0.0001	
Yes	31 (91.2)	3 (8.8)	34 (100)		
Total	125 (95.4)	6 (4.6)	131(100)		
Bone Pain during past month					
Yes	18 (37.5)	30 (62.5)	48 (100)	0.0011	
No	3 (50.0)	3 (50.0)	6 (100)		
Total	21 (38.9)	33 (61.1)	54 (100)		
Bone Crises since last submission					
Yes	15 (83.3)	3 (16.7)	18 (100)	0.0001	
No	29 (100.0)	0	29 (100)		
Total	44 (93.6)	3 (6.4)	47 (100)		
	10 years following first infusion,* N* (%)				
First infusion	Mild	Moderate	Severe	Total	
Thrombocytopenia (platelet count x 10^3^/mm^3^)					
Mild (≥120)	120 (99.2)	1 (0.8)	0	121 (100)	0.9201
Moderate (60 to <120)	13 (100.0)	0	0	13 (100)	
Severe (<60)	7 (100.0)	0	0	7 (100)	
Total	140 (99.3)	1 (0.7)	0	141 (100)	
Hepatomegaly					
Mild (≤1.25)	6 (100.0)	0	0	6 (100)	0.0273
Moderate (>1.25 to ≤2.5)	16 (88.9)	2 (11.1)	0	18 (100)	
Severe (>2.5)	4 (50.0)	4 (50.0)	0	8 (100)	
Total	26 (81.3)	6 (18.8)	0	32 (100)	

*SD* standard deviation

^a^Anemia is defined according to age and gender norms for hemoglobin concentrations, as follows: < 12 g/dl for males >12 years; < 11 g/dl for females >12 years; < 10.5 g/dl for children ages > 2 –12 years; < 9.5 g/dl for children ages 6 months to 2 years; < 10.1 g/dl for children <6 months

*Tests of null hypotheses (i.e., no change from first infusion to 10 years) were calculated using the McNemar test

**Tests of the null hypotheses (i.e., no change from first infusion to 10 years) were calculated using the chi-square test

Changes in hematological, visceral organ, and skeletal characteristics for splenectomized patients reported at first infusion and after 10 years of treatment are shown in Table 3. The patients demonstrated a significant (*P* < 0.0001) improvement in mean hemoglobin concentration (g/dl) 10 years following first infusion (from 11.9 to 13.4). In fact, 91.2 % of patients who were anemic at first infusion were no longer so after 10 years of treatment (Table 5, Supplementary Fig. 1b). Platelet counts improved with treatment (mean at baseline 237.8 × 10^3^/mm^3^ vs. mean at 10 years 311.2 × 10^3^/mm^3^), as it did nonsplenectomized patients (Tables 3 and 5, Supplementary Fig. 2b). The number of nonsplenectomized patients with severe thrombocytopenia decreased significantly (*P* < 0.0001) after 10 years; no splenectomized patients had severe thrombocytopenia at 10 years (Table 5, Supplementary Fig. 2b). Liver volumes significantly improved (*P* < 0.001) in splenectomized patients after 10 years (Table 3), with mean volume decreasing from 2.2 MN to a median of 1.0 MN (Table 3). The response was similar to that of nonsplenectomized patients, who had less hepatomegaly prior to treatment (mean at first infusion 1.8 MN decreased to a mean of 1.0 MN at 10 years). The percentage of splenectomized patients reporting bone pain at first infusion was 88.9 % (48/54), which decreased to 61.1 % (33/54) after 10 years (Table 5, Supplementary Fig. 5b). The majority of patients without bone pain after 10 years were from the initial subgroup reporting bone pain (18/21, 85.7 %). Absence of bone pain prior to first infusion was reported for only six splenectomized patients, three of whom reported bone pain when evaluated after 10 years. Compared with nonsplenectomized patients, higher percentages reported bone pain prior to and after 10 years, irrespective of bone pain status prior to treatment. Treatment positively affected the frequency of bone crises in splenectomized patients (Table 5, Supplementary Fig. 6b), decreasing from 38.3 % (18/47) to 6.4 % (3/47), all of whom were from the subgroup reporting bone crises prior to treatment. Similar to nonsplenectomized patients, at the start of treatment, the majority of splenectomized patients received imiglucerase in the middle two dose ranges: >15 to ≤45 U/kg every 2 weeks (*N* = 74, 37.0 %); >45 to ≤90 U/kg every 2 weeks (*N* = 75, 37.5 %) (Table 6). The remainder (*N* = 51, 25.5 %) received low doses (≤15 U/kg every 2 weeks), and no patient received high doses (>90 to ≤150 U/kg every 2 weeks). After 10 years, the majority of patients (*N* = 107, 53.5 %) were receiving imiglucerase doses in the range of 15 to ≤45 U/kg every 2 weeks, reflecting a shift from other dose groups. The dose distribution at 10 years appeared similar to that of nonsplenectomized patients.

**Table 6:** Distribution of dose received^a^ at first infusion and at 10 years following first infusion for patients with type 1 Gaucher disease,* N* (%)

Dosage distribution						
Nonsplenectomized^b^	10 years following first infusion					
First infusion dose received (U/kg every 2 weeks)	≤15	>15 ≤45	>45 ≤90	>90 ≤150	Total	*P* value
≤15	54 (48.2)	46 (41.1)	11 (9.8)	1 (0.9)	112 (100)	<0.0001
>15 ≤45	29 (11.9)	154 (63.1)	61 (25.0)	0	244 (100)	
>45 ≤90	12 (6.1)	120 (60.6)	63 (31.8)	3 (1.5)	198 (100)	
>90 ≤150	0	1 (33.3)	2 (66.7)	0	3 (100)	
Total	95 (17.1)	321 (57.6)	137 (24.6)	4 (0.7)	557 (100)	
Splenectomized	10 years following first infusion					
First infusion dose received (U/kg every 2 weeks)	≤15	>15 ≤45	>45 ≤90	>90 ≤150	Total	*P* value
≤15	22 (43.1)	26 (51.0)	2 (3.9)	1 (2.0)	51 (100)	<0.0001
>15 ≤45	9 (12.2)	45 (60.8)	20 (27.0)	0	74 (100)	
>45 ≤90	5 (6.7)	36 (48.0)	32 (42.7)	2 (2.7)	75 (100)	
>90 ≤150	0	0	0	0	0	
Total	36 (18.0)	107 (53.5)	54 (27.0)	3 (1.5)	200 (100)	

^a^Frequencies and percentages along the diagonal self-explanatory--> represent patients whose dose group did not change from first infusion to 10 years following initiation of therapy. Those above the diagonal indicate patients whose dose increased, and those below the diagonal patients whose dose decreased

^b^Includes 27 patients with partial splenectomy

*Tests of the null hypotheses (i.e., no change from first infusion to 10 years) were calculated using the chi-square testtable-->

## Discussion

The focus of this study, which analyzed the responses of GD1 patients at first infusion and after 10 years of imiglucerase treatment, was to assess whether responses to therapy were maintained for this time period. Clinical responses, including the influence of spleen status, were analyzed in a large cohort of nonsplenectomized (*n* = 557) and splenectomized (*n* = 200) GD1 patients enrolled in the ICGG Gaucher Registry. The results indicated that all parameters studied responded favorably to imiglucerase after 10 years of treatment.

The ICGG Gaucher Registry is a voluntary enterprise, and all registry data are analyzed retrospectively and unaudited by the study authors (Hollak et al. 2011). Data on each parameter are not available for every patient, but the collective data set far exceeds any previously described. The ICGG Gaucher Registry has made diligent efforts to verify data as complete and accurate. Patients analyzed in this study are clinically heterogeneous. Once patients have achieved stable disease parameters, their monitoring investigation schedules are generally less intensive, and regular organ imaging does not occur after 8–10 years of treatment, resulting in the relatively small numbers for some parameters in our analysis. Nevertheless, the ICGG Gaucher Registry offers the possibility of evaluating a very large sample of patients with GD1.

Although the relationship between long term effectiveness data and the occurrence of safety and adverse event data are of interest, these were not included in this analysis because data collected through Genzyme’s pharmacovigilance system cannot be linked to data in the registry. Safety and adverse event data among Cerezyme users have been previously published and reviewed (Starzyk et al. 2007; Elstein and Zimran 2009).

The patients in this 10-year response study constitute 34.5 % (*N* = 767) of 2,222 registry patients with GD1 with whom alglucerase or imiglucerase was initiated in 1999 or earlier (35.9 % of all patients with intact spleens and 29.7 % of all total splenectomy patients). This study’s patients are indistinguishable from the larger registry population in terms of gender, age at diagnosis, and age of first infusion (Supplementary Table 1). Additionally, regardless of splenectomy status, the difference between age at first infusion and at last registry data entry for all 2,222 GD1 patients is 10–12 years, suggesting there is little if any selection bias due to attrition of more severely affected, poorly responding patients. The lack of relevant data at the prescribed 10-year data point was the overwhelming reason for exclusion. Of the 2,222 GD1 patients who began alglucerase/imiglucerase in 1999 or earlier, 149 died [71 of 1,549 (5 %)] intact spleen; 78 of 673 (12 %) total splenectomy]. The adequacy of response to treatment in these deceased patients will be evaluated as part of an in-progress mortality analysis.

Splenectomy influences the degree of cytopenia caused by hypersplenism (Hughes et al. 2007) is a recognized risk factor for avascular osteonecrosis (Deegan et al. 2011; Mistry et al. 2009) and is associated with arterial and venous thrombosis and pulmonary arterial hypertension (Crary and Buchanan 2009). Since the advent of imiglucerase therapy, the need for splenectomy to control signs and symptoms of GD1, including severe cytopenias and abdominal discomfort, has been substantially reduced (Cox et al. 2008). To account for the effects of splenectomy on patients with GD1, we analyzed the cohort in two groups. Similar numbers of patients in both groups (nonsplenectomized 47 %; splenectomized 48 %) were diagnosed with GD1 before 10 years of age. The mean age at diagnosis for these two groups was 18 and 15 years, respectively, which is similar to some previous registry reports (Charrow et al. 2000; Weinreb et al. 2002) but younger than a single-center case series and treatment study (Grabowski et al. 1995; Zimran et al. 1992). The differences between reports may be due to local genotypic and diagnostic differences, or alternatively, possible selection and reporting bias of registry data (Rothman 2002).

After 10 years of imiglucerase, approximately 90 % of all patients who were anemic at first infusion had normalized hemoglobin levels. We were unable to determine from the registry data whether anemia at the 10-year point was due to refractory GD or some alternative etiology. At this same time point, >90 % (6/7) of patients who had severe thrombocytopenia at first infusion had improved platelet counts. Despite consistent improvements in thrombocytopenia in patients with intact spleens, proportionately fewer patients had normal platelet counts after 10 years of treatment than patients who had normal hemoglobin concentrations. This observation is consistent with earlier studies of imiglucerase (Weinreb et al. 2002), velaglucerase (Zimran et al. 2010), and taliglucerase (Zimran et al. 2011) and is attributable to multiple possible causes. These causes include differences in platelet and red blood cell (RBC) kinetics; different pooling mechanisms in the spleen; different control mechanisms in the marrow and marrow microenvironment for erythropoiesis and thrombopoiesis, chronic platelet activation, and consumption due to bone disease; differences in the effect of changes in plasma volume on RBC versus platelet concentrations in peripheral blood; presence of antiplatelet antibodies in some patients; and different sensitivity of RBC and platelets to reduction in spleen size (Hollak et al. 2012). All splenectomized patients with thrombocytopenia at first infusion had normal counts at 10 years. The enhanced response in splenectomy patients also reflects previous experience and likely reflects the major contribution of the spleen to the thrombocytopenia due to GD and the relative ease of reversibility of depressed thrombopoiesis in GD-infiltrated marrow. Of patients with severe splenomegaly at first infusion, 97 % had decreased spleen volumes after 10 years. Similar results were reported for liver volumes: all patients who had severe hepatomegaly at first infusion had decreased liver volumes after 10 years.

Genotypes of most of GD1 patients in this study were N370S homozygous or heterozygous, with one of the following mutations: L444P, rare allele, 84GG, or IVS2 + 1. Whereas the L444P/L444P genotype was reported in a few patients (8/493 nonsplenectomized; 4/189 splenectomized), all of those patients were reported by their physicians as having a GD1 phenotype.

Pretreatment hematological and visceral organ abnormalities reported in this study were generally similar to those previously reported by Charrow et al. (2000). Although blood and visceral organ abnormalities are potentially important contributors to morbidity in patients with GD1, hepatosplenomegaly and hematological cytopenias are generally correctable, save for a relatively small number of patients with pretreatment complications, such as fibrotic spleen or liver fibrosis and portal hypertension. On the other hand, skeletal manifestations associated with untreated GD1 have the potential to cause long-term disability and negatively impact the patient’s quality of life permanently (Weinreb et al. 2007). In this study, by the time treatment was begun, bone pain was reported by the majority of nonsplenectomized (98/187) and nearly all splenectomized (48/54) patients. Bone crises, which often signal irreversible osteonecrosis, were reported not only in splenectomized (18/47) patients, but also in those with intact spleens (27/169). The percentage of splenectomized patients with bone pain and bone crises was approximately twice that of nonsplenectomized patients (89 % vs 52 % and 38 % vs 16 %, respectively). Because of the inevitable occurrence of irreversible damage, delay in initiating treatment in patients with bone complications is, as we found, likely to be reflected in a poorer long-term response and clinical outcome.

Previous reports (Grabowski et al. 1998; Pastores et al. 1993; Weinreb et al. 2002) describe the effects of alglucerase/imiglucerase treatment after 6 months to 5 years. Pastores et al. (1993) and Weinreb et al. (2002) reported improvements in all hematological and visceral organ parameters within the first 6 months to 2 years of therapy. Specifically, Weinreb et al. (2002) reported that patients with anemia achieved normal hemoglobin levels within 2 years of starting imiglucerase, and hemoglobin levels were sustained or increased during the initial 5 years of therapy. In nonsplenectomized patients, the level of platelet response was associated with degree of thrombocytopenia (Weinreb et al. 2002), presence of focal splenic defects (Stein et al. 2010), and dose of imiglucerase (Grabowski et al. 2009). Generally, platelet counts increased during the first 2 years of treatment and continued to improve through 5 years (Weinreb et al. 2002). Similarly, Weinreb et al. (2002) reported decreased spleen and liver volumes with imiglucerase treatment: spleen volumes decreased for the first 2 years, whereas reductions in liver volume may continue to year 5 (Weinreb et al. 2002). Weinreb et al. (2002) noted that liver volumes generally normalized, but decreases in spleen volume to less than five times normal rarely occurred.

A previous report (Wenstrup et al. 2007) indicated that skeletal manifestations, including bone density, took longer (≥8 years) to respond to imiglucerase than other hematological and visceral organ manifestations of GD1. Many patients in our study responded positively to imiglucerase treatment, as noted by reported reductions in bone crises and bone pain. Of the nonsplenectomized patients reporting pain at first infusion, 93 % (25/27) and 57 % (56/98) reported no bone crises nor bone pain, respectively, after 10 years. Decreased bone pain and bone crisis have been reported after the first year of imiglucerase treatment (Charrow et al. 2007; El-Beshlawy et al. 2006; Weinreb et al. 2002). Of the splenectomized patients reporting pain at first infusion, after 10 years, 38 % (18/48) reported no bone pain and 83 % (15/18) no bone crises. Splenectomy has been reported to be a risk factor for the skeletal manifestations of GD1 (Deegan et al. 2011; Mistry et al. 2009). An interval of ≥2 years between diagnosis and initiation of imiglucerase therapy was associated with a higher risk of severe skeletal complication of avascular osteonecrosis (Mistry et al. 2009). It is possible that other skeletal complications are also related to the time between diagnosis and the start of therapy.

Reports of bone pain to the registry might not necessarily be attributable to GD and may not necessarily be reversible. In addition, it may either be overreported to the registry as limb pain not emanating from bony complications, or misreported as skeletal involvement not related to GD. Similarly, bone crises may be exaggerated if the definition is not strictly adhered to by registry physicians. Such variations may have occurred at first infusion and 10 years after imiglucerase treatment. Because our analysis focused on only two points over a 10-year observation period (first infusion and 10 years), bone pain results may not accurately portray the patient’s chronic state. Despite this possible reporting bias, there was a decrease in bone pain and bone crisis symptoms after 10 years of therapy. Nevertheless, some patients with chronic bone pain may continue to be symptomatic even after years of therapy. Some of these patients may potentially benefit from modern, expert orthopedic interventions.

The clinical responses reported in this study were observed over a broad range of imiglucerase doses at initiation of therapy and at the 10-year period. Response to imiglucerase is statistically related to treatment dose (Grabowski et al. 2009; Wenstrup et al. 2007), although clinically, patients responded over a wide range of doses. In general, after 10 years of therapy, biweekly dose was reduced compared with initial dose. Patients may change dose over time for a number of reasons, including adequate response, compliance, and enzyme availability.

In summary, improvements in hematological, visceral, and bone manifestations previously reported after short-term imiglucerase therapy were maintained after 10 years of treatment in nonsplenectomized and splenectomized patients with GD1.

## Electronic supplementary material

Below is the link to the electronic supplementary material.

Figure 1 Change in Anemia from First Infusion of Imiglucerase to 10 Years in A. Non-Splenectomized and B. Splenectomized Type 1 Patients (*p* < 0.0001) (JPEG 36 kb)

High resolution image file (TIF 963 kb)

Figure 2 Change in Thrombocytopenia from First Infusion of Imiglucerase to 10 Years in A. Non-Splenectomized (*p*<0.0001) and B. Splenectomized Type 1 Patients (*p*=0.9201) (JPEG 46 kb)

High resolution image file (TIF 1288 KB)

Figure 3 Change in Splenomegaly from First Infusion of Imiglucerase to 10 Years in Non-Splenectomized Type 1 Patients (*p* < 0.0001) (JPEG 23 kb)

High resolution image file (TIF 16917 KB)

Figure 4 Change in Hepatomegaly from First Infusion of Imiglucerase to 10 Years in A. Non-Splenectomized (*p*<0.0001) and B. Splenectomized Type 1 Patients (*p* = 0.0273) (JPEG 44 kb)

High resolution image file (TIF 31712 KB)

Figure 5 Change in Bone Pain from First Infusion of Imiglucerase to 10 Years in A. Non-Splenectomized (*p*<0.0001) and B. Splenectomized Type 1 Patients (*p* = 0.0011) (JPEG 38 kb)

High resolution image file (TIF 970 KB)

Figure 6 Change in Bone Crisis from First Infusion of Imiglucerase to 10 Years in A. Non-Splenectomized and B. Splenectomized Type 1 Patients (*p* < 0.0001) (JPEG 34 kb)

High resolution image file (TIF 835 KB)

Supplementary Table 1 (DOC 53.5 kb)

## Footnotes

1 In 2011, Genzyme Corporation became Genzyme, a Sanofi company.
