# Supplementary figures and images for: Long-term clinical outcomes in type 1 Gaucher disease following 10 years of imiglucerase treatment
Source: J Inherit Metab Dis. 2012 Sep 14;36(3):543–53. doi: 10.1007/s10545-012-9528-4 (PMC3648688; doi:10.1007/s10545-012-9528-4)

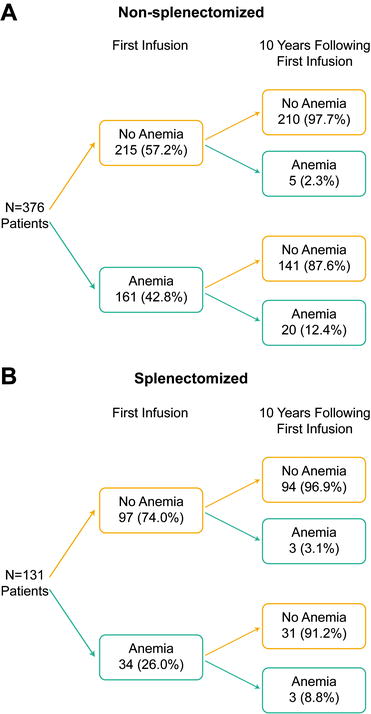

Supplement: Supplementary file 1 — Change in Anemia from First Infusion of Imiglucerase to 10 Years in A. Non-Splenectomized and B. Splenectomized Type 1 Patients (p < 0.0001) (JPEG 36 kb) [file 10545_2012_9528_Fig1_ESM.jpg]

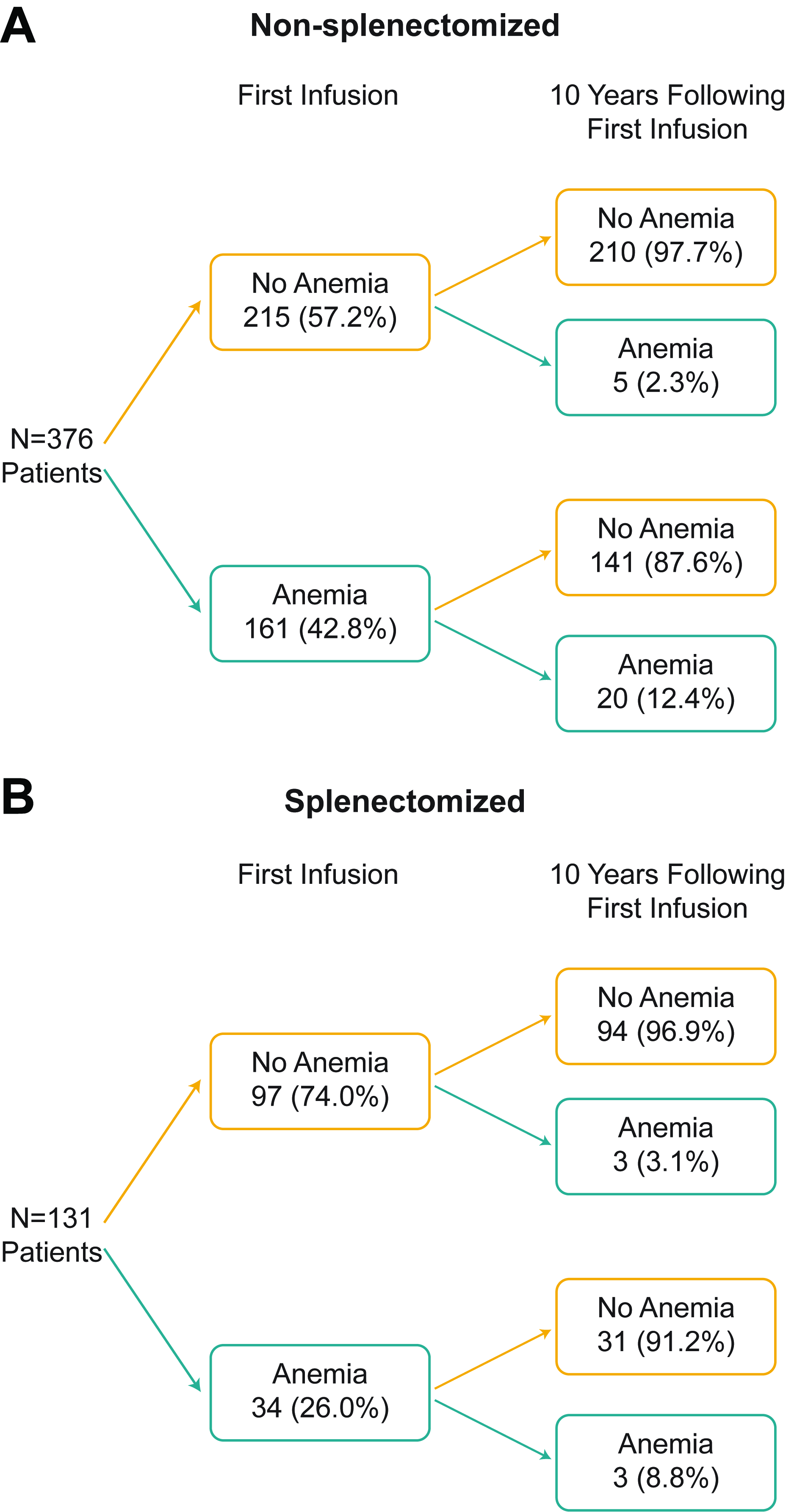

Supplement: Supplementary file 2 — High resolution image file (TIF 963 kb) [file 10545_2012_9528_MOESM1_ESM.tif]

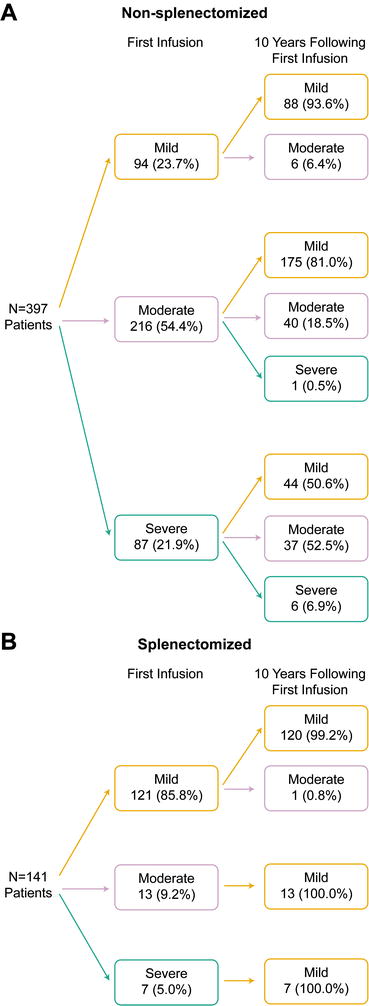

Supplement: Supplementary file 3 — Change in Thrombocytopenia from First Infusion of Imiglucerase to 10 Years in A. Non-Splenectomized (p<0.0001) and B. Splenectomized Type 1 Patients (p=0.9201) (JPEG 46 kb) [file 10545_2012_9528_Fig2_ESM.jpg]

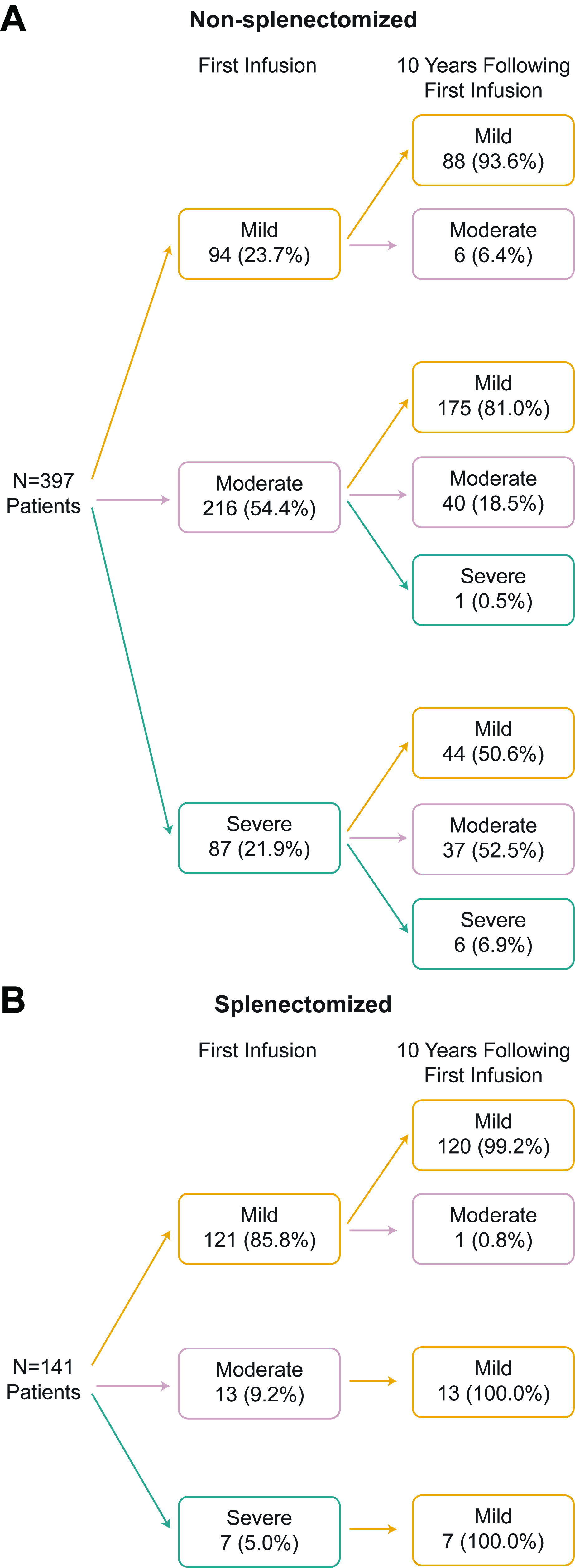

Supplement: Supplementary file 4 — High resolution image file (TIF 1288 KB) [file 10545_2012_9528_MOESM2_ESM.tif]

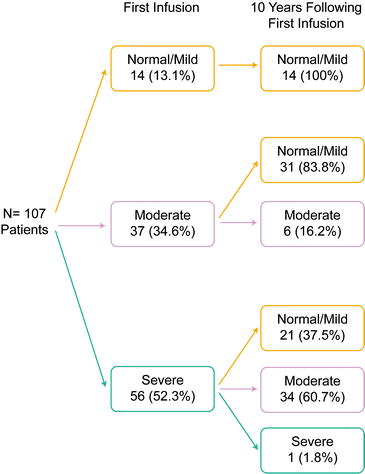

Supplement: Supplementary file 5 — Change in Splenomegaly from First Infusion of Imiglucerase to 10 Years in Non-Splenectomized Type 1 Patients (p < 0.0001) (JPEG 23 kb) [file 10545_2012_9528_Fig3_ESM.jpg]

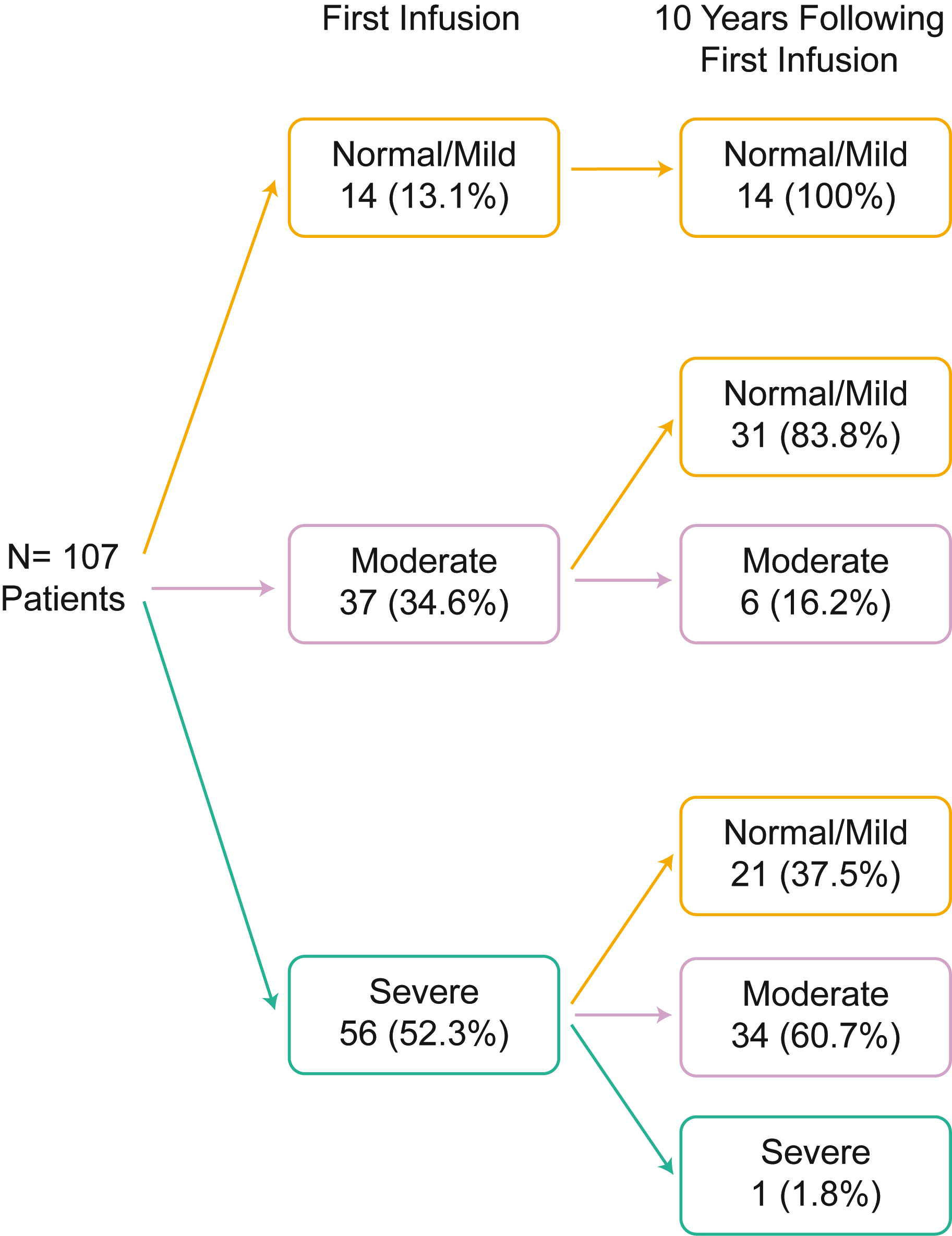

Supplement: Supplementary file 6 — High resolution image file (TIF 16917 KB) [file 10545_2012_9528_MOESM3_ESM.tif]

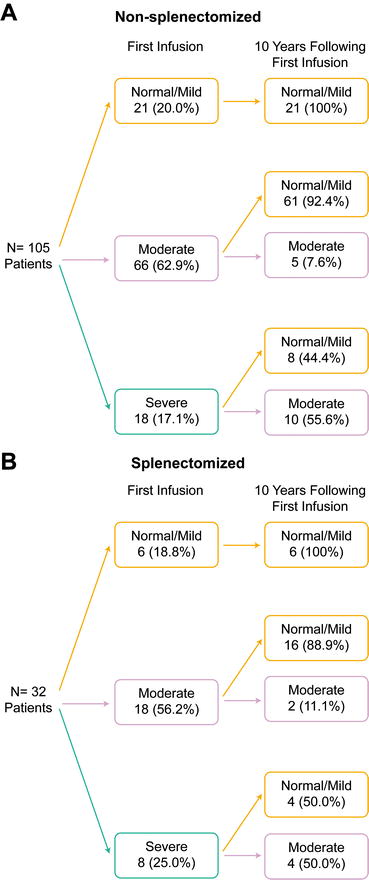

Supplement: Supplementary file 7 — Change in Hepatomegaly from First Infusion of Imiglucerase to 10 Years in A. Non-Splenectomized (p<0.0001) and B. Splenectomized Type 1 Patients (p = 0.0273) (JPEG 44 kb) [file 10545_2012_9528_Fig4_ESM.jpg]

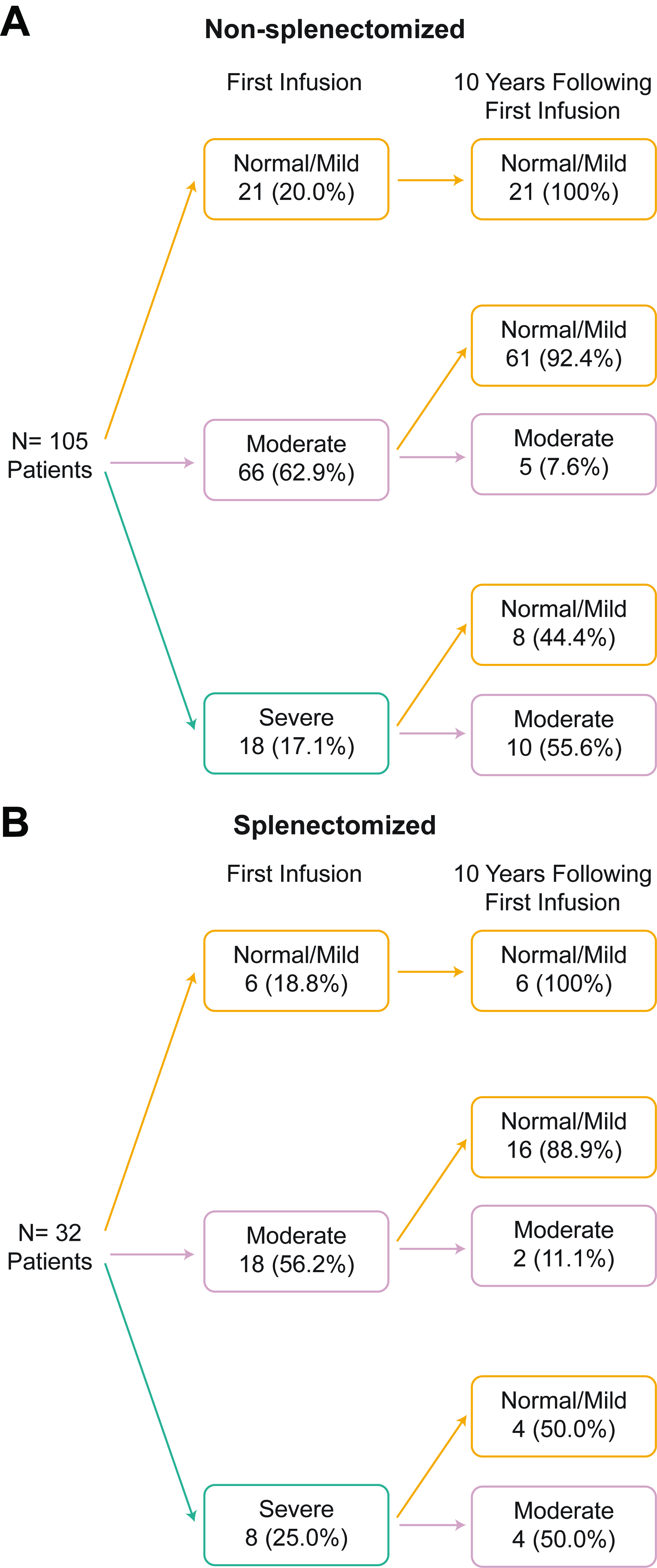

Supplement: Supplementary file 8 — High resolution image file (TIF 31712 KB) [file 10545_2012_9528_MOESM4_ESM.tif]

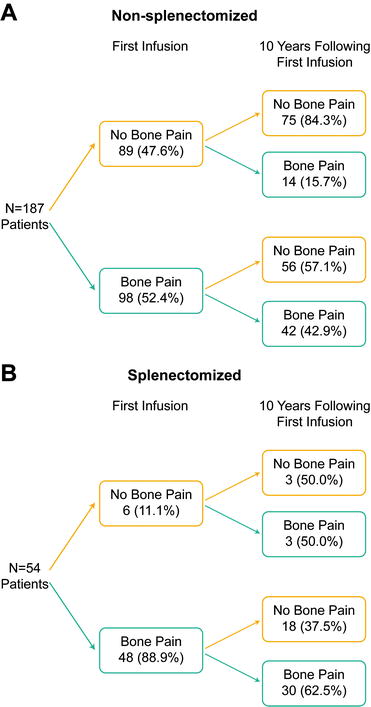

Supplement: Supplementary file 9 — Change in Bone Pain from First Infusion of Imiglucerase to 10 Years in A. Non-Splenectomized (p<0.0001) and B. Splenectomized Type 1 Patients (p = 0.0011) (JPEG 38 kb) [file 10545_2012_9528_Fig5_ESM.jpg]

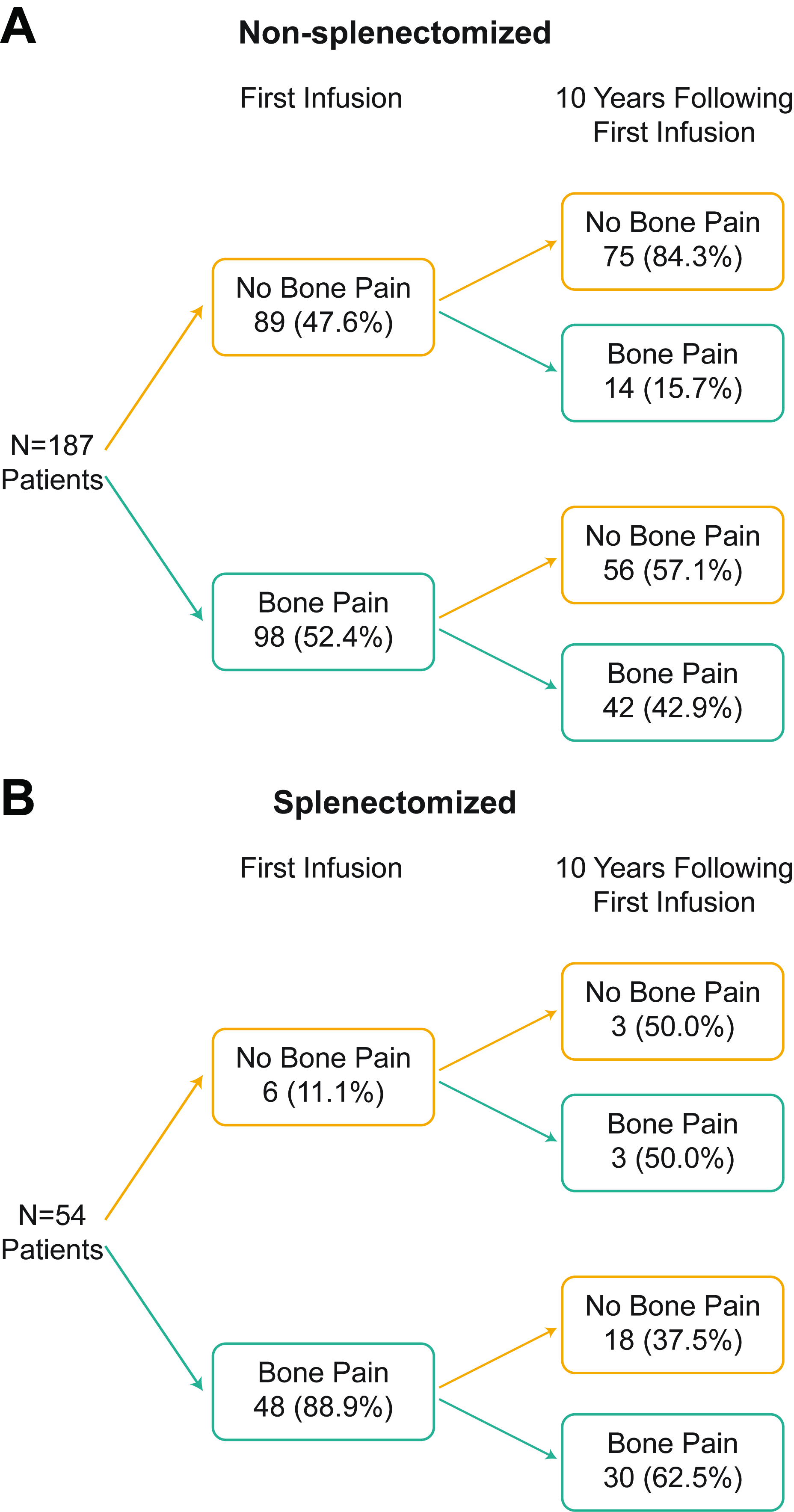

Supplement: Supplementary file 10 — High resolution image file (TIF 970 KB) [file 10545_2012_9528_MOESM5_ESM.tif]

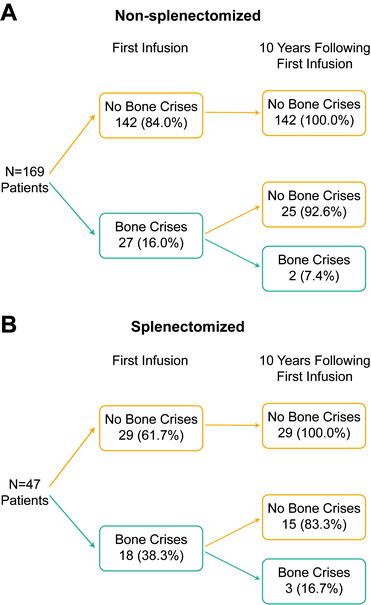

Supplement: Supplementary file 11 — Change in Bone Crisis from First Infusion of Imiglucerase to 10 Years in A. Non-Splenectomized and B. Splenectomized Type 1 Patients (p < 0.0001) (JPEG 34 kb) [file 10545_2012_9528_Fig6_ESM.jpg]

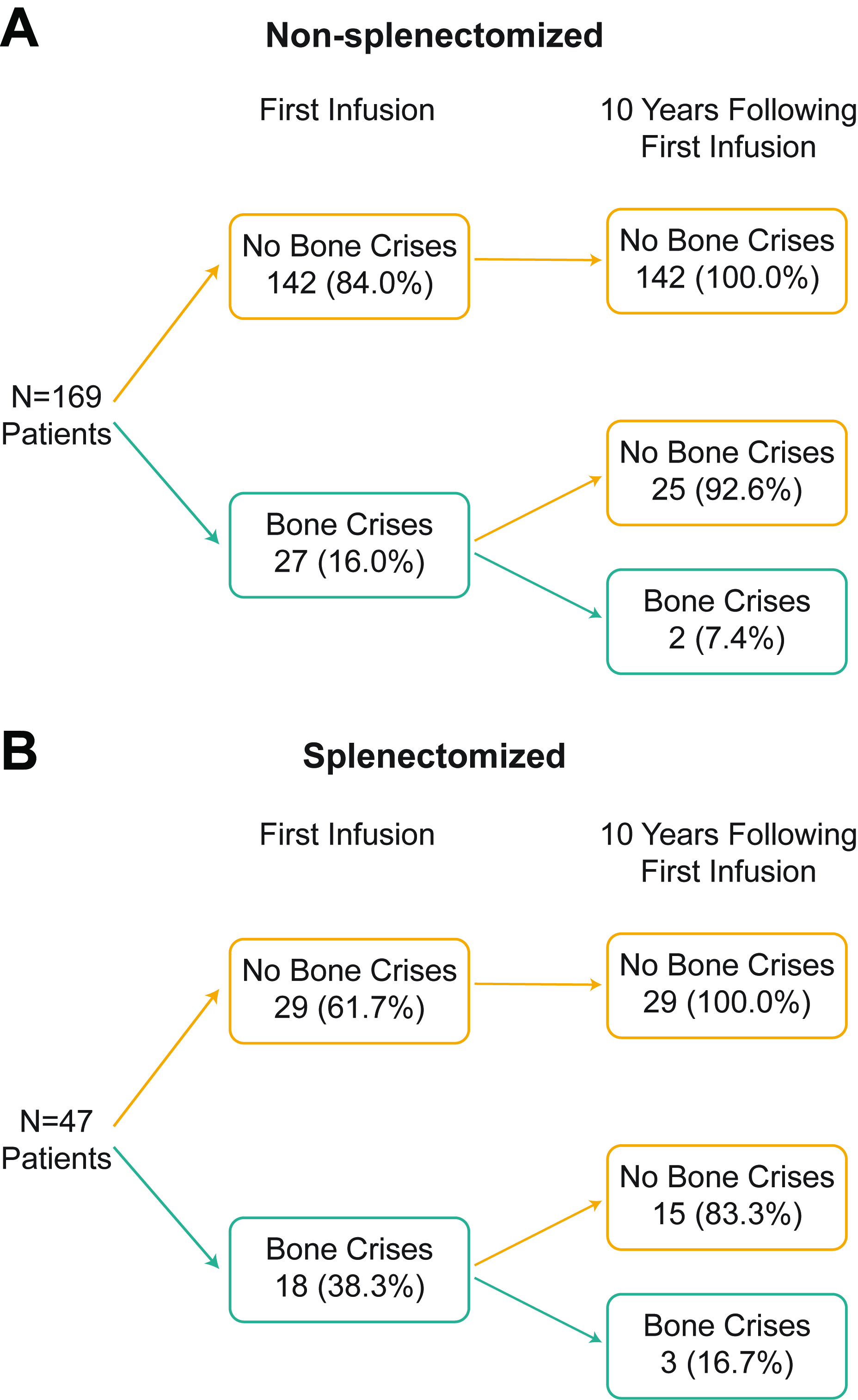

Supplement: Supplementary file 12 — High resolution image file (TIF 835 KB) [file 10545_2012_9528_MOESM6_ESM.tif]
