# Supplementary material for: Long-term clinical outcomes in type 1 Gaucher disease following 10 years of imiglucerase treatment
Source: J Inherit Metab Dis. 2012 Sep 14;36(3):543–53. doi: 10.1007/s10545-012-9528-4 (PMC3648688; doi:10.1007/s10545-012-9528-4)
Supplement: Supplementary file 13 — (DOC 53.5 kb) [file 10545_2012_9528_MOESM7_ESM.doc]

| **Supplementary Data Table 1**  **Patient Characteristics for all Type 1 Patients Treated with Alglucerase/Imiglucerase with First infusion in 1999 or earlier** | | |
| --- | --- | --- |
|  | **Non-splenectomized** | **Splenectomized** |
| **Patients Enrolled** | **1,549** | **673** |
| ***Sex, n (%)*** | ***1,549*** | ***673*** |
| Males | 732(47) | 286(42) |
| Females | 817(53) | 387(58) |
| ***Age at Diagnosis^†^ (y)*** | ***1484*** | ***633*** |
| Median (25th, 75th) | 13( 5, 32) | 12( 5, 24) |
| Mean (SD) | 20(18) | 16(14) |
| Min, Max | -2, 83 | -7, 78 |
| ***Age at Diagnosis^†^, n (%)*** | ***1,484*** | ***633*** |
| Prenatal^‡^ to <10 Years | 636(43) | 287(45) |
| 10 to <20 Years | 234(16) | 134(21) |
| 20 to <30 Years | 217(15) | 117(18) |
| 30 to <40 Years | 165(11) | 52( 8) |
| 40 to <50 Years | 117( 8) | 29( 5) |
| 50 to <60 Years | 58( 4) | 6( 1) |
| 60 to <70 Years | 38( 3) | 3( 0) |
| 70 Years or More | 19( 1) | 5( 1) |
| ***Age at First Infusion (y)*** | ***1549*** | ***673*** |
| Median (25th, 75th) | 25(11, 42) | 37(26, 48) |
| Mean (SD) | 28(20) | 37(16) |
| Min, Max | 0, 85 | 1, 80 |
| ***Age at Last Follow-up (y)*** | ***1549*** | ***673*** |
| Median (25th, 75th) | 35(23, 54) | 48(36, 60) |
| Mean (SD) | 39(20) | 48(16) |
| Min, Max | 1, 92 | 4, 89 |
| Reported Deceased, n (%)  No | 1,478(95) | 595(88) |
| Yes | 71( 5) | 78(12) |

^†^Patients with no diagnosis date or with diagnosis date earlier than 1 year prior to

birth were excluded from the analysis.

^‡^ Diagnosed prenatally.
